# Supplementary material for: Bioinformatic Analysis for Influential Core Gene Identification and Prognostic Significance in Advanced Serous Ovarian Carcinoma
Source: Medicina (Kaunas). 2021 Sep 4;57(9):933. doi: 10.3390/medicina57090933 (PMC8470004; doi:10.3390/medicina57090933)
Supplement: Supplementary file 1 [file medicina-57-00933-s001.zip › Supple table 1.pdf]

Table S1. *CDCA3* mRNA expression levels in relation to clinicopathological parameters of TCGA OV.

Parameters		*CDCA3* expression levels		Chi-square	P value
		Higher (N=154)	Lower (N=154)		
Age	<60	80	89	1.062	0.303
	≥60	74	65		
	Null	0	0		
Clinical stage	I and II	11	12	0.047	0.828
	III and IV	142	141		
	Null	1	1		
Histologic grade	GB, G1, G2	13	27	5.437	0.020
	G3, G4	137	125		
	Null	4	2		
Lymphatic invasion	No	23	21	0.020	0.889
	Yes	45	39		
	Null	86	94		
Venous invasion	No	23	19	0.025	0.875
	Yes	31	24		
	Null	100	111		
Primary therapy outcome	CR, PR, SD	97	107	1.059	0.303
	PD	13	9		
	Null	44	38		
Personal tumor status	Tumor free	51	48	0.134	0.714
	With tumor	103	106		
	Null	0	0		
Vital status	Alive	58	66	0.864	0.353
	Deceased	96	88		
	Null	0	0		
