# Supplementary material for: Bioinformatic Analysis for Influential Core Gene Identification and Prognostic Significance in Advanced Serous Ovarian Carcinoma
Source: Medicina (Kaunas). 2021 Sep 4;57(9):933. doi: 10.3390/medicina57090933 (PMC8470004; doi:10.3390/medicina57090933)
Supplement: Supplementary file 1 [file medicina-57-00933-s001.zip › Supple table 2.pdf]

Table S2. *UBE2C* mRNA expression levels in relation to clinicopathological parameters of TCGA OV.

Parameters		*UBE2C* expression levels		Chi-square	P value
		Higher (N=154)	Lower (N=154)		
Age	<60	81	88	0.642	0.423
	≥60	73	66		
	Null	0	0		
Clinical stage	I and II	21	11	0.034	0.854
	III and IV	142	141		
	Null	0	2		
Histologic grade	GB, G1, G2	14	26	4.150	0.042
	G3, G4	137	125		
	Null	3	3		
Lymphatic invasion	No	23	21	0.395	0.530
	Yes	39	45		
	Null	92	88		
Venous invasion	No	24	18	1.302	0.254
	Yes	25	30		
	Null	105	106		
Primary therapy outcome	CR, PR, SD	101	103	0.002	0.965
	PD	11	11		
	Null	42	40		
Personal tumor status	Tumor free	56	43	2.516	0.113
	With tumor	98	111		
	Null	0	0		
Vital status	Alive	63	61	0.054	0.816
	Deceased	91	93		
	Null	0	0		
